# Effect of Tendon Vibration on Hemiparetic Arm Stability in Unstable Workspaces

**DOI:** 10.1371/journal.pone.0144377

**Published:** 2015-12-03

**Authors:** Megan O. Conrad, Bani Gadhoke, Robert A. Scheidt, Brian D. Schmit

**Affiliations:** 1 Department of Industrial and Systems Engineering, Oakland University, Rochester, Michigan, United States of America; 2 Department of Biomedical Engineering, Marquette University, Milwaukee, Wisconsin, United States of America; VU University Amsterdam, NETHERLANDS

## Abstract

Sensory stimulation of wrist musculature can enhance stability in the proximal arm and may be a useful therapy aimed at improving arm control post-stroke. Specifically, our prior research indicates tendon vibration can enhance stability during point-to-point arm movements and in tracking tasks. The goal of the present study was to investigate the influence of forearm tendon vibration on endpoint stability, measured at the hand, immediately following forward arm movements in an unstable environment. Both proximal and distal workspaces were tested. Ten hemiparetic stroke subjects and 5 healthy controls made forward arm movements while grasping the handle of a two-joint robotic arm. At the end of each movement, the robot applied destabilizing forces. During some trials, 70 Hz vibration was applied to the forearm flexor muscle tendons. 70 Hz was used as the stimulus frequency as it lies within the range of optimal frequencies that activate the muscle spindles at the highest response rate. Endpoint position, velocity, muscle activity and grip force data were compared before, during and after vibration. Stability at the endpoint was quantified as the magnitude of oscillation about the target position, calculated from the power of the tangential velocity data. Prior to vibration, subjects produced unstable, oscillating hand movements about the target location due to the applied force field. Stability increased during vibration, as evidenced by decreased oscillation in hand tangential velocity.

## Introduction

Stroke survivors are prone to instability when arm movements are made in planar [1,2] or three-dimensional workspaces [3]. Previously, we examined how vibratory stimuli applied to the flexor tendons of the forearm affect hand trajectory during horizontal planar point-to-point movements [1]. In these prior studies we found that for hemiparetic stroke survivors, vibration of the flexor tendons of the hemiparetic wrist improve stability of the paretic arm immediately after movement. Although the primary trajectories of point-to-point movements were similar with and without vibration, the ability to stabilize the hand about the desired final position was improved during and after tendon vibration for movements in some directions. The largest effects of vibration occurred in directions associated with the greatest observed instability at the shoulder. In the current study, we sought to determine whether tendon vibration of the forearm musculature could improve stabilization in both stroke and healthy subjects during point-to-point movements in an unstable force field.

Although the specific mechanisms of action remain largely unknown, interventions that stimulate sensory afferents have demonstrated potential to improve motor performance post-stroke. For example, electric stimulation, applied as a general stimulus to the wrist prior to a motor task, improves motor function in the arm by enhancing activity in sensorimotor pathways and motor output from the human cortex [4,5]. Vibration is also effective in stimulating sensory afferents in order to improve motor performance. Vibration, applied as a subsensory stimulus (i.e. below the sensory threshold) to the foot improves posture and balance in older adults, patients with diabetes, and individuals with stroke [6,7]. Muscle or tendon vibration is a powerful means of stimulating proprioceptive afferents [8,9] and is capable of enhancing activity in many of the same neuromotor pathways believed responsible for functional improvements elicited by electric stimulation [10]. Additionally, vibration is known to enhance sensory influence on cortical motor control systems [11], suggesting that vibration has potential to alter limb movement control, perhaps by altering central processing.

In the arm, multi-joint spinal [12] and supraspinal [13, 14] reflexes are well documented and believed to be exaggerated after stroke [15, 16]. Exaggerated reflex activity may occur either as a direct or indirect result of damage to the sensorimotor cortex. Because tendon vibration has the capacity to enhance cortical influence on motor systems [10, 11] through increased activity in the sensorimotor cortex [17, 18], we hypothesized that tendon vibration applied to wrist flexor tendons could improve shoulder stability through multi-joint spinal pathways.

## Materials and Methods

### Subject Population

Ten chronic stroke survivors (6 female, 4 male; age range 44–63; mean age, 55.5) and 5 age-matched neurologically-intact control subjects (3 female, 2 male; age range 50–61; mean age, 56.2) participated in this study. Stroke survivors were required to be more than 1 year post-stroke and to experience upper-extremity hemiparesis. Prior to experimentation, the impairment level of stroke survivors was measured by the upper extremity Fugl-Meyer Assessment (FMA: maximum score = 66) [19]. Stroke survivors reported being between 1 and 22 years post-stroke and FMA scores ranged from 21–63. Subjects were excluded if unable to give informed consent, experienced cognitive dysfunction that precluded comprehension of the experiment, had a diagnosis of other neuromuscular disorder or had recently (< 3 months) used botulinum toxin or other substances interfering with neuromuscular function. Control subjects were required to have no history of stroke or pathology of the upper extremity. Pilot data indicated less variability within control subject data due to the force field. As a result, fewer control subjects were tested than stroke patients. The study and consent procedures were approved by the Marquette University Institutional Review Board (IRB). All subjects gave written, informed consent in accord with the Helsinki Declaration.

### Experimental Protocol

**Fig 1:**
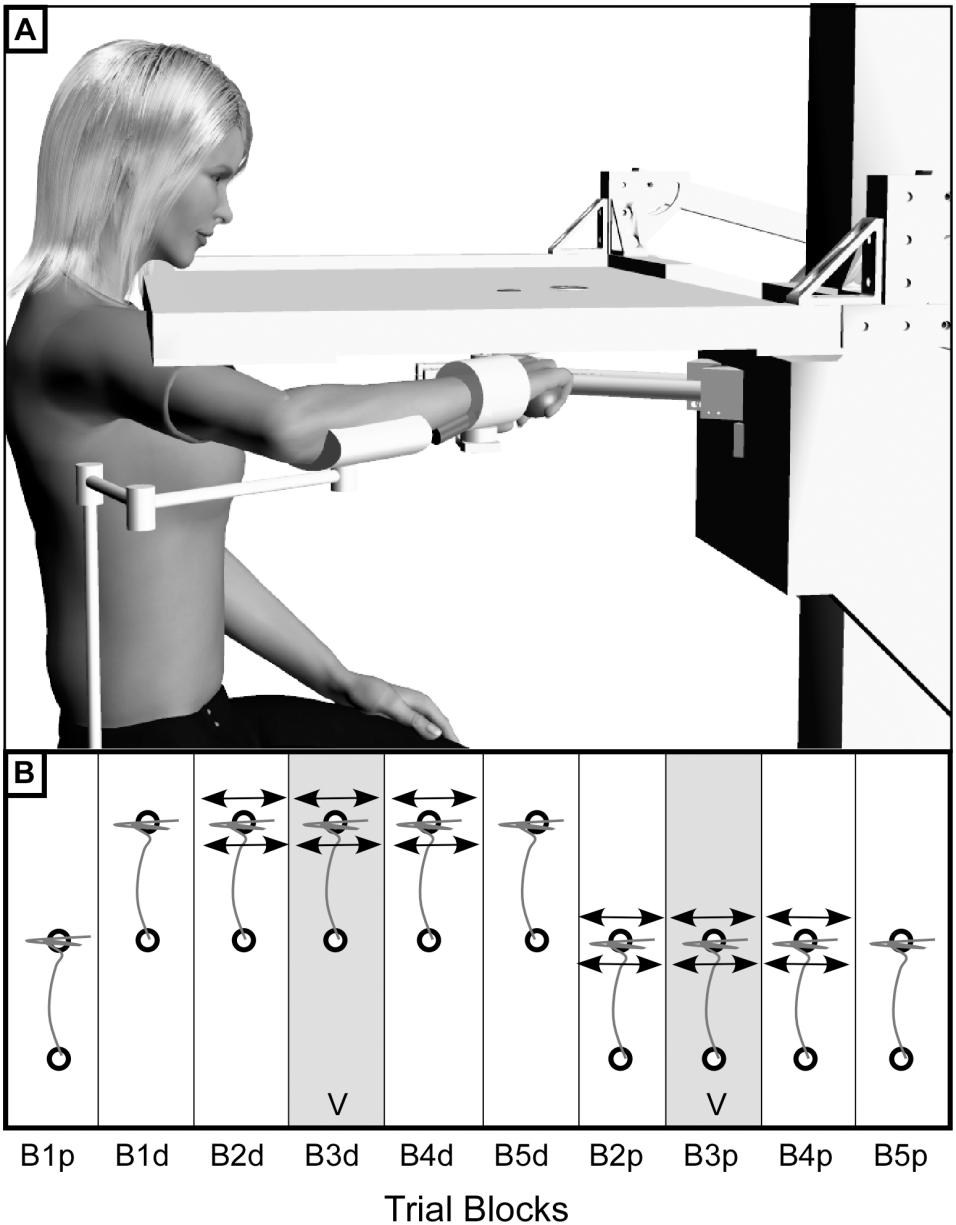
Experimental Set-Up. **A)** Planar arm movements were made while each subject grasped the handle of a two-joint planar robot. Optical encoders provided measurements of position data, which was converted to a global x (medial/lateral) and y (forward/backward) coordinate system. Tasks were projected onto a horizontal screen, which obstructed the subject’s view of their hand and arm. The subject’s arm was supported by an armrest and the base of the pronated wrist was attached to the robot’s manipulandum with a wrist brace. Grip pressure was measured by a pressure bladder, which was placed in the subject’s palm. Subject depicted in figure is a computer generated image and is in no way representative of any actual subject. **B)** Twenty trials were conducted in each experimental condition totaling 200 trials. Initially, two blocks of trials were conducted to quantify baseline movement in proximal and distal workspace. Then in one workspace (proximal or distal) a block of trials was conducted measuring the response to a divergent force field (F_d_). Tendon vibration was applied in the following block, in addition to F_d_, to determine the effect of vibration on the ability to stabilize the hand at the end of movement. Aftereffects of vibration were evaluated during a block of trials with only F_d_, which was followed by another block of baseline trials. The treatment and washout blocks were then repeated in the other (proximal or distal) workspace.

Subjects were seated in front of a robot that allowed arm movement only in the horizontal plane (Fig 1A) [1, 20]. Stroke subjects completed the experiment with their hemiparetic arm while the control group used their dominant arm. A lightweight armrest supported the arm against gravity. A high backed chair with chest straps constrained trunk movement. A horizontal screen obstructed direct view of the hand and forearm. The base of the subject’s hand was attached to the robot manipulandum at the wrist via a rigid brace such that the position of the manipulandum changed with the location of the wrist. This allowed the subject to grasp a pressure bladder that measured the force of grip during movement. The robot measured wrist position and velocity, interface forces arising between the subject's wrist and the robot's end-effector as well as grip force. As described previously [21] the robot could produce force fields that were able to assist or resist movement within the planar workspace. Tendon vibration was accomplished using a custom computer-controlled vibrator placed beneath the wrist brace so that it pressed firmly against the wrist flexor tendons. The tendon vibrator consisted of an offset mass that rotated about a motor shaft (Faulhaber Group, Clearwater, FL) at a low amplitude (<1mm) and frequency. The apparatus was encased by a Teflon sleeve, which protected subjects from contact with the revolving mass.

Surface electrodes were placed on the arm and forearm in accordance with previously published sensor placement recommendations [22] to collect electromyographic (EMG) signals using a commercially available EMG amplifier (Bortec Medical AMT-16; Calgary, Alberta, Canada). The skin over the muscle belly of the wrist flexors (WF; flexor carpi radialis), wrist extensors (WE; extensor carpi ulnaris), brachioradialis (BRD), biceps (BI), triceps (TRI), anterior deltoid (AD), posterior deltoid (PD) and pectoralis major (PEC) was cleaned and lightly abraded before attaching the disposable Ag/AgCl electrodes (Vermed Medical, Bellows Falls, VT). The EMG signals were amplified (500x or 1000x) and band-pass filtered (10 to 330 Hz) prior to sampling at 1000 Hz. Maximum voluntary contractions (MVCs) were recorded during a series of six isometric, 3 s maximal-effort exertions into wrist flexion and extension, elbow flexion and extension, and shoulder flexion and extension as the experimenter resisted the subject's movements. Later, maximal EMG readings were used to normalize trial EMG data for comparison across subjects.

The position of the hand beneath the opaque screen was represented by a red cursor (0.5 cm dia.), which was projected onto the screen above the wrist. Prior to the experiment each subject’s active range of motion was tested as they gripped the manipulandum to ensure each subject could reach both the proximal and distal target. Subjects performed 200 goal-directed arm movements (called *trials*). Movements were organized into 5 blocks of 20 trials in each of two workspaces. *Proximal workspace* movements started from a point 10 cm anterior to the sternum. *Distal workspace* movements started from a point 24 cm anterior to the sternum. In each trial, the subject moved the cursor from a circular home target (1 cm dia.) to a goal target (1 cm dia.) located 14 cm further from the body in the sagittal plane. The beginning of a trial was signaled by the appearance of a target on the screen and was accompanied by an auditory tone. When a target appeared, subjects were instructed to move the cursor as quickly and accurately as possible to the target, where they were to stabilize the cursor for an additional 3 s. At the end of the trial, the targets disappeared and the robot moved the arm back to the home position in preparation for the next movement. Table 1 summarizes the characteristics of each block of trials.

**Table 1:** Experiment Block Conditions.

*Block Name*	*Characteristics*	*Number of Trials*
Block 1 (B1)	No Force, No Vib	40
Block 2 (B2)	Force	40
Block 3 (B3)	Force + Vib	40
Block 4 (B4)	Force	40
Block 5 (B5)	No Force, No Vib	40

The first block of trials in each workspace (proximal: B1_p_; distal: B1_d_) was used to measure nominal, baseline performance of goal-directed reaching movements. Next, a divergent force field was applied to the second (B2), third (B3) and fourth (B4) blocks in each workspace. During B3 blocks, 70 Hz vibration was applied continuously to the forearm wrist and finger musculature, allowing measurement of the effect of vibration on limb stabilization within the unstable divergent force field that was applied at the end of movement. Aftereffects of vibration were measured in block B4, wherein only the force field was applied. The fifth block (B5) contained no forces or vibration and was used to evaluate residual effects of both treatments on the baseline motion. Trial blocks took ~ 5 minutes each to complete.

**Fig 2:**
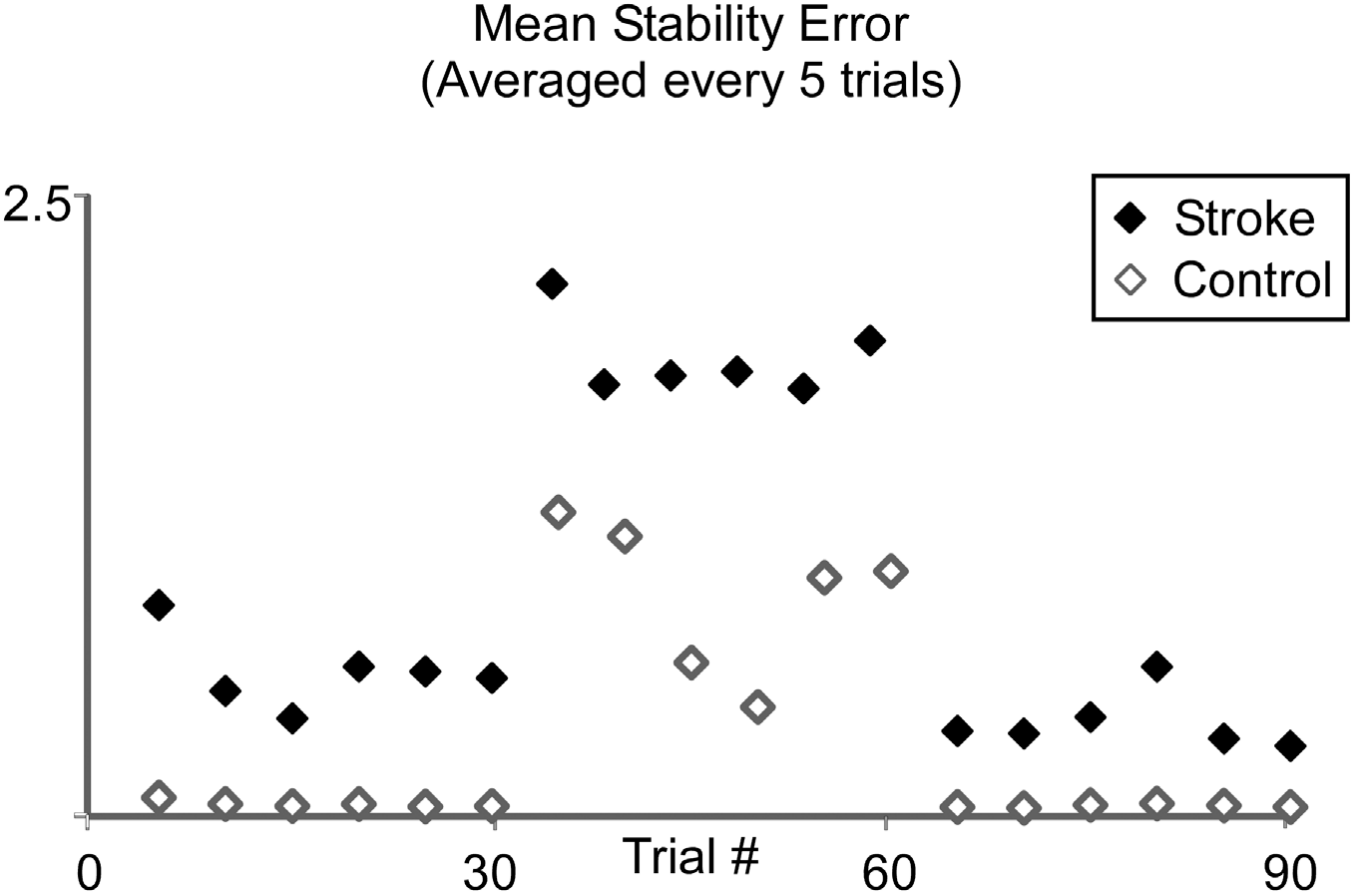
Pilot Study Results: Adaptation to Divergent Force Field. Plots of mean stability error (averaged every 5 trials) observed across stroke and control subjects during a pilot experiment aimed at selecting force parameters. Each subject made 90 forward movements from the home to the target location in the proximal workspace. In the initial 30 trials, no force field was present in order to assess baseline performance. The following 30 trials included the force field (Control G = 40Ns/m; Stroke G = 20 Ns/m). The final 30 trials again were without the force field to investigate kinematic after-effects.

The divergent force field (F_d_) applied in B2, B3 and B4 was chosen to accentuate endpoint instability in the medial-lateral direction (i.e., the component associated primarily with shoulder joint movement). Medial/lateral forces were applied within a 10 cm (medial-lateral) x 6 cm (anterior-posterior) rectangular window centered on the goal target:

$$F_{d}=\left[\begin{array}{c} F_{x}\\ F_{y} \end{array}\right]=G\left[\begin{array}{cc} 1 & 0\\ 0 & 0 \end{array}\right]\left[\begin{array}{c} \dot{x}\\ \dot{y} \end{array}\right]$$ (1)

where *F*_*x*_ and *F*_*y*_ are the components of force in the x (medial-lateral) and y (anterior-posterior) directions, *G* is a constant gain factor, and *ẋ* and *ẏ* are the medial/lateral and proximal/distal components of hand velocity, respectively. The gain assigned to stroke subjects was 20 Ns/m, while the gain for controls was 40 Ns/m. These gain values were based on pilot data collected from each subject (Fig 2), which indicated that these values produced unstable movement patterns of approximately the same magnitude in both subject groups. For the pilot testing, subjects made reaching movement between the home and target locations. No force field was present in the first and last 30 trials whereas the middle trials included the imposed force fields (Control: G = 40Ns/m; Stroke: G = 20 Ns/m). All subjects were able to overcome the field F_d_ and stabilize the cursor within +/- 5 cm of the target, such that the final positions attained at the end of trials in the middle block were similar to those in before and after perturbation (see Fig 2). At the specified gains however, F_d_ was strong enough to exaggerate terminal oscillations about the target as revealed by plots of mean stability error, which is a measure of the magnitude of hand oscillations in the neighborhood of the goal target. Subjects were unable to compensate for the divergent force field even after 30 trials of exposure.

Fig 1B shows the sequence of trial blocks performed by half of the subjects. The remaining subjects reversed the workspace presentation order to account for potential order effects. The initial baseline blocks in each workspace were performed prior to imposing the divergent field and vibratory treatments in order to avoid the possibility that treatments in one workspace might confound baseline performance in the other workspace.

### Data Analysis

Instantaneous hand position data were low pass filtered using a second-order, zero-lag Butterworth filter with a 20-Hz cutoff before computing tangential velocity (hand speed). Recent experimental evidence demonstrates that in reaching, the brain invokes a feedforward control action to initiate limb movement toward the goal and a separate control action to terminate movement and stabilize the hand at its final position [23]. We partitioned reach trajectories into distinct movement and stabilization phases. The movement trajectory phase started at the time of *movement onset* (t_s_; defined as the first time at which hand speed surpassed 20% of its maximum value) and ended when the hand dropped below 20% of that peak velocity (*end of movement*: t_e_). We considered movement duration to span t_s_ to t_e_. A stabilization phase included all motions following t_e_. Analyses of performance during the stabilization phase focused on the period from t_e_ to t_e_ + 1 s. Endpoint stability at the target was quantified by two performance measures derived from the power spectral density of the hand’s tangential velocity during the hold period. *Stability error* (S_e_) was defined as the magnitude of hand oscillation about the target location and was estimated as the area under the power spectrum curve between 1 Hz and 5 Hz [1]. *Error frequency* (f_e_) was defined as the frequency at which half of the area under the power spectrum lie on either side of that frequency during the stabilization period.

#### Muscle activity

EMG data analysis focused on the 1 s stabilization period at the end of movement. EMG signals underwent bandpass filtering (10–350 Hz) and notch filtering to eliminate power line noise (59–61 Hz) and mechanical artifacts resulting from the vibratory stimulus (68–72 Hz and the first harmonics 136–144 Hz). In each case, zero-phase, second order Butterworth filters were used. The root-mean square (RMS) values of the movement and MVC trial data were calculated using a 100 ms sliding window. The resulting movement trial EMG time series from each muscle were divided by their respective peak MVC RMS values and then integrated within the stabilization period to yield an estimate of each muscle's contribution to stabilization.

#### Grip pressure

A second order, low pass, Butterworth filter (5 Hz cutoff) was used to filter grip pressure data obtained from the pressure transducer. The change in grip pressure during each trial was calculated by subtracting the initial value of grip pressure from its peak value.

#### Statistical analysis

A repeated-measures multivariate analysis of variance (MANOVA) was run to determine the effect of subject group (control, stroke), experimental block (B1, B2, B3, B4, and B5) and workspace location (proximal, distal) on stability error, error frequency, and the trial mean increase in grip pressure. The significant effect of group and experimental condition led to the performance of univariate ANOVAs to further investigate the effect of these two factors, with the results collapsed across the two workspace locations. Tukey post hoc tests were used to identify differences among the five experimental block conditions. A separate MANOVA was run to test the effect of vibration on the amplitude of muscle activity for each recorded muscle.

## Results

**Fig 3:**
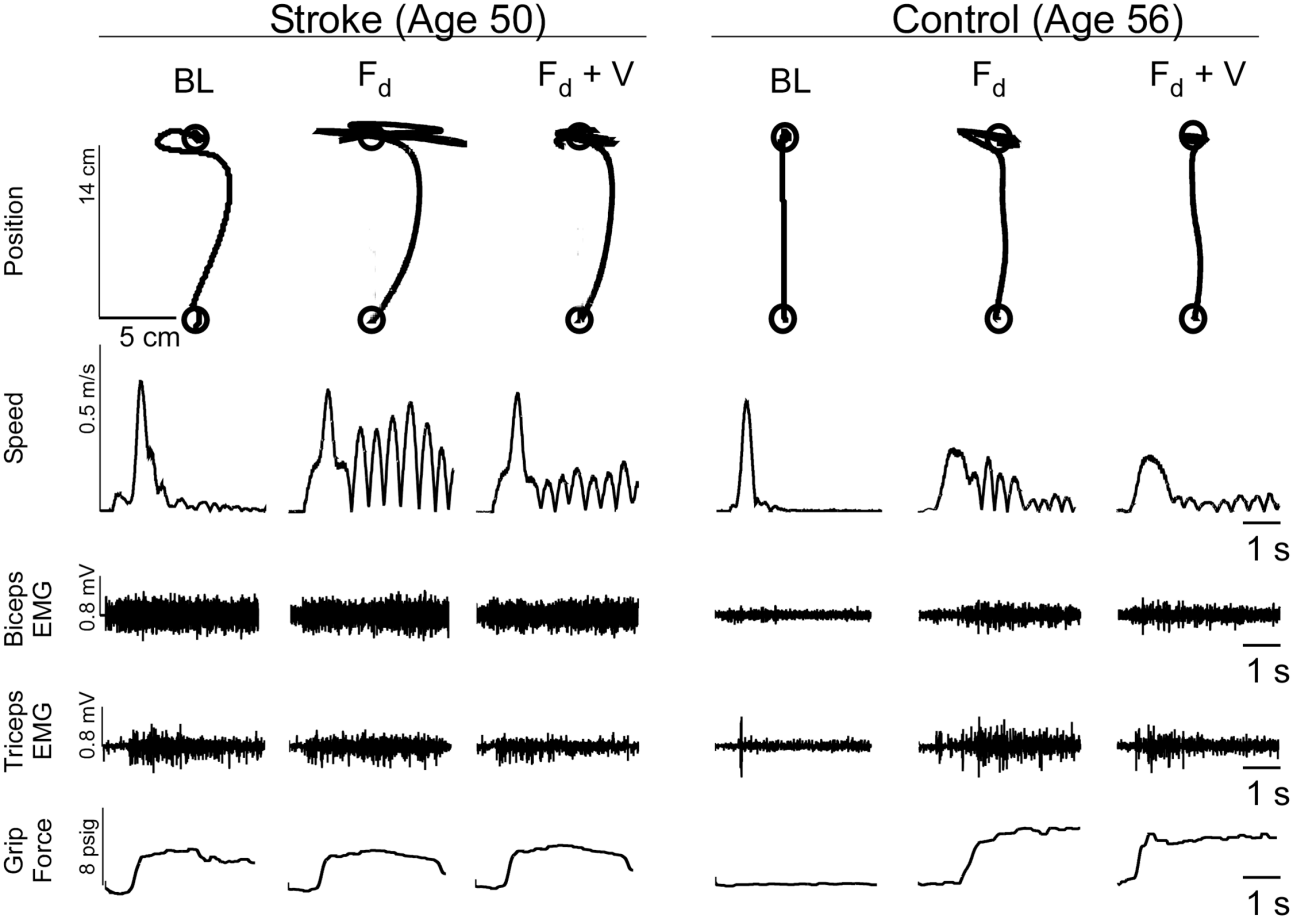
Characteristic Movement. Plots of typical position and velocity profiles representing arm movements made by a chronic stroke and an age-matched neurologically intact control subject while making arm movements at baseline, into a divergent force field (F_d_), and into F_d_ with vibration (V). Compared to baseline trials, F_d_ increased instability at the target position. Tendon vibration appeared to improve stability at the target for both subjects as evidenced by decreased movement at the target position and decreased oscillation amplitudes in the tangential velocity data during the stabilization period. EMG data indicated that control subjects co-contract antagonist muscles and increase their grip in response to F_d_. Stroke subjects, whose EMG and grip activity was already heightened during baseline trials were unable to invoke the same compensation.

We sought to quantify the effects of wrist tendon vibration on endpoint stability at the end of reaching movements made by stroke survivors and neurologically-intact control subjects. A divergent force field was implemented in the neighborhood of the goal target so that both groups of subjects experienced difficulty in stabilizing their hand at the end of movement. While reaching in the force field, motion in the medial/lateral direction of F_d_ was apparent for all subjects trying to stabilize at the end of the movement (Fig 3). However, compared to stroke survivors, the control group generally took a more direct path to the target and experienced less instability (or smaller oscillatory movements) in the medial/lateral direction after reaching the goal. Plots of the hand's tangential velocity revealed that both groups invariably made an initial primary movement followed by smaller secondary motions to reach the target (Fig 3). The magnitude of these secondary movements decreased with tendon vibration, indicating greater endpoint stability.

An initial MANOVA revealed a main effect of Group (F = 57.438, p < 0.001) and Block (F = 5.50; p < 0.001), as well as an interaction between these factors (F = 1.995; p = 0.024), with significant effects on the three primary performance measures {stability error, error frequency, grip pressure}. Subsequent univariate ANOVAs were performed on each dependent variable revealed a significant Group effect for stability error (F = 3.032, p < 0.001) and error frequency (F = 10.092, p < 0.001); a significant Block effect for stability error (F = 12.722, p < 0.001), error frequency (F = 4.106, p = 0.004), and grip pressure (F = 2.639, p = 0.037); and a significant Group*Block effect on error frequency (F = 3.740, p = 0.006) and grip pressure (F = 10.092, p < 0.001). Results of post-hoc Tukey tests for significant contrasts are reported in Tables 2, 3 and 4.

**Table 2:** Statistical significance of changes in stability error (S_e_) observed between experimental blocks.

	Block 2	Block 3	Block 4	Block 5
	Force	Force + Vib	Force	
***Stroke*** (n = 10)				
Block 1	0.006*	0.964	0.994	0.914
Block 2: Force		0.040*	0.019*	<0.001*
Block 3: Force + Vib			0.999	0.557
Block 4: Force				0.721
***Control*** (n = 5)				
Block 1	< 0.001*	0.436	0.020*	1.000
Block 2: Force		0.003*	0.133	< 0.001*
Block 3: Force + Vib			0.564	0.423
Block 4: Force				0.019*

* p-values obtained from Tukey post hoc analysis of S_e_ data (α = 0.05).

**Table 3:** Statistical significance of changes in error frequency observed between experimental blocks.

	Block 2	Block 3	Block 4	Block 5
	Force	Force + Vib	Force	
***Stroke*** (n = 10)				
Block 1	0.945	0.786	0.986	1.000
Block 2: Force		0.995	0.722	0.980
Block 3: Force + Vib			0.474	0.874
Block 4: Force				0.958
***Control*** (n = 5)				
Block 1	0.011*	0.003*	< 0.001*	0.548
Block 2: Force		0.985	0.721	0.326
Block 3: Force + Vib			0.948	0.126
Block 4: Force				0.023*

* p-values obtained from Tukey post hoc analysis of error frequency data (α = 0.05).

**Table 4:** Statistical significance of changes in grip pressure observed between experimental blocks.

	Block 2	Block 3	Block 4	Block 5
	Force	Force + Vib	Force	
***Stroke*** (n = 10)				
Block 1	0.631	0.845	0.999	0.919
Block 2: Force		0.996	0.468	0.177
Block 3: Force + Vib			0.707	0.349
Block 4: Force				0.978
***Control*** (n = 5)				
Block 1	0.033*	0.025*	0.040*	0.472
Block 2: Force		1.000	1.000	0.646
Block 3: Force + Vib			1.000	0.577
Block 4: Force				0.695

* p-values obtained from Tukey post hoc analysis of grip pressure data (α = 0.05).

**Fig 4:**
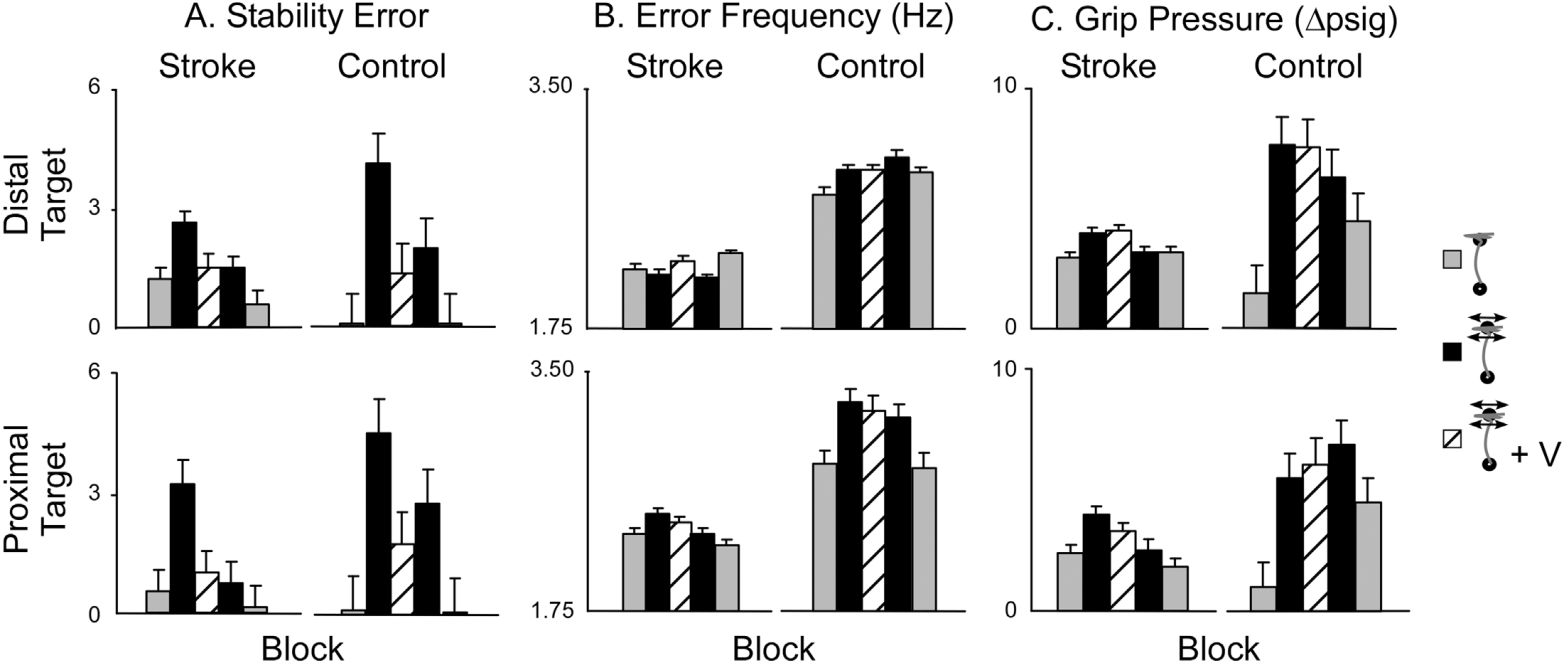
Effect of Vibration on Arm Movements. **A)** Stability error (mean ± SD) reported at proximal and distal targets for stroke (n = 10) and control (n = 5) subjects. Stability error increased for both subject groups while stabilizing in F_d_. Tendon vibration decreased stability error. **B)** The frequency of hand oscillation (Hz, mean ± SD) at the target was not significantly affected by the F_d_ or tendon vibration. However, control subjects exhibited significantly higher error frequencies than the stroke group. **C)** Changes in grip pressure (psig, mean ± SD) across blocks of trials as subjects made movements at proximal and distal target locations. Control subjects increased their grip pressure while attempting to stabilize their hand during movements made in F_d_. Stroke subjects did not demonstrate significant increases in grip pressure while stabilizing in F_d_.

A comparison of stability error across groups indicated that at baseline, stroke subjects had greater difficulty stabilizing at the target compared to the control group (Fig 4). Trials performed in the force field elicited higher instability for both stroke and control groups compared to baseline trials. Vibration of the wrist tendon resulted in an improvement in stability for both groups. Despite working within the same force field, post-vibration improvements in stability remained lower than pre-vibration trials on average. In the final set of trials, when the force field was removed, both groups performed better than during the baseline trials. Table 2 summarizes the paired comparisons for stability error between experimental conditions for both subject groups.

The mean error frequency across all blocks was significantly higher for control subjects compared to the stroke group in both the proximal and distal workspaces (Fig 4B). Table 3 summarizes the paired comparisons calculated for error frequency between experimental conditions for both subject groups. The destabilizing force field induced an increase in error frequency for control subjects only and only at the proximal target. Vibration yielded no significant decrease of error frequency for either group.

The mean change in grip pressure exerted on the hand-held bladder is reported in Fig 4C. During all trials, stroke subjects demonstrated a tendency to tighten their grip as they moved their hand away from the body. On average, stroke subjects initially exerted higher grip pressure on the bladder throughout each trial than did control subjects (see representative data, Fig 3). During baseline trials, control subjects rarely exerted pressure on the pressure bladder while stroke subjects consistently tightened their grip as the hand moved away from the torso. In response to F_d_, the control group responded to the field by tightening their grip on the pressure bladder. For these control subjects, grip pressure remained significantly higher than the baseline average for the remainder of trials (see Table 4). The stroke group did not significantly increase grip pressure during trials made in F_d_ as their grip pressure during baseline trials was already relatively high. Fig 4 depicts the mean change in grip pressure (pre-movement to movement) for stroke and control subjects at both the proximal and distal target locations. Table 4 summarizes the paired comparisons calculated for grip pressure between experimental conditions for each subject groups.

**Fig 5:**
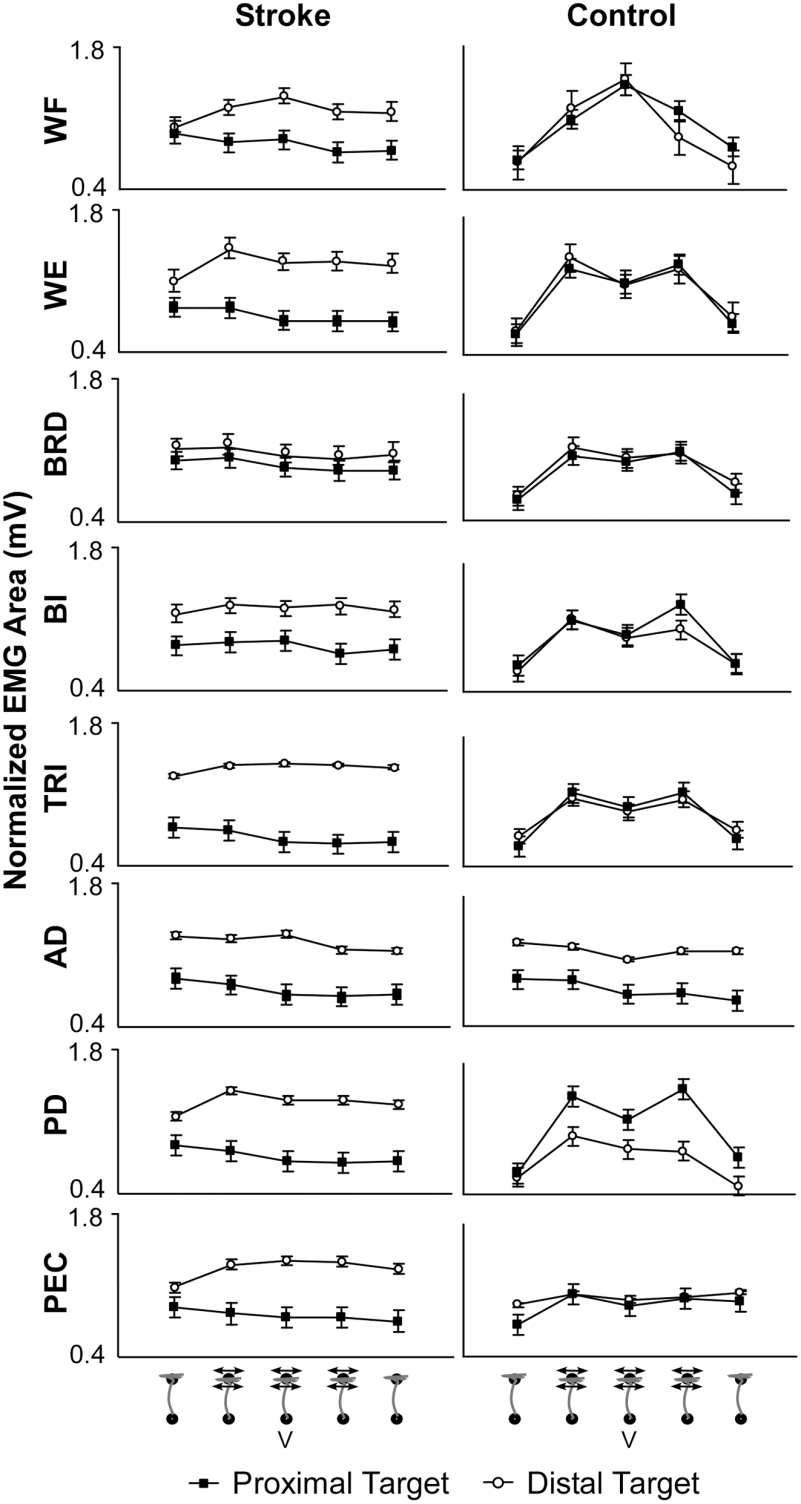
EMG Data. Normalized EMG (mean ± SD) area are shown for stroke and control subjects’ wrist flexors (WF), wrist extensors (WE), brachioradialis, (BRD), biceps (BI), triceps (TRI), anterior deltoid (AD), posterior deltoid (PD), and pectoralis major (PEC) muscles. Stroke subjects always exhibited higher muscle activation levels at distal targets. Control subjects used relatively similar levels of muscle activation at each target for all but the AD (greater in distal workspace) and PD (greater in proximal workspace) muscles. Control subjects also increased muscle activity in response to F_d_.

A repeated measures MANOVA was run to test the effect of Block and target location on EMG data. Normalized EMG data revealed that control subjects significantly increased the magnitude of muscle activity in WF, WE, BRD, BI, TRI, and PD muscles in response to F_d_ (post-hoc ANOVA; p < 0.05). Stroke subjects did not produce similar increases in muscle activity in response to the divergent force field. For the control group, muscle activity in the WF, WE, BRD, BI, TRI and PEC was similar whether working in the proximal or distal workspace. However, control subjects working in the distal workspace required higher AD activity, while stability in the proximal workspace relied more heavily on the PD. For stroke subjects, higher levels of muscle activity were always experienced while working in distal workspaces (post-hoc ANOVA; p < 0.001). Fig 5 portrays the difference in muscle activity between stroke and control subjects during each set of trial conditions. The mean magnitude of EMG activity did not significantly change in any muscle group in response to vibration.

## Discussion

We have demonstrated that wrist tendon vibration can positively influence hand stability during arm movements made in divergent force fields. Initially, both stroke and control subjects experienced high levels of instability with the application of a divergent force field. In addition to heightened instability, control subjects exhibited a tendency to increase grip pressure and muscle activity throughout the arm in response to F_d_. However, stroke subjects did not demonstrate the same levels of heightened grip pressure and increased arm muscle activation in response to the force field. The application of vibration significantly improved endpoint stability. This effect was not a simple training effect because no similar reduction in stability error was observed during pilot testing, even after 30 trials of exposure to the same magnitudes of perturbation used in our primary study (Fig 2). Thus, the results indicate that improvements in endpoint stability recorded at the hand can be elicited by wrist tendon vibration in both stroke as well as age-matched control subjects moving in this unstable environment.

### Response to Divergent Force Field and Tendon Vibration

In the control group, a comparison of B1 to B2 trial block performance indicated that neurologically intact individuals increased muscle activity in 6 of the 8 muscles in response to destabilization of the hand. Increased muscle activation was accompanied by an average increase in grip pressure across trials made in B2 (initial perturbation block). These results are consistent with prior reports that the healthy CNS uses a strategy of impedance control, selectively co-contracting pairs of agonist/antagonist muscles, to attain increased endpoint stiffness in unstable work environments [24–28]. Unlike previous studies, however, the strength of F_d_ in this experiment was intentionally set sufficiently high such that full adaptation to the field was not possible. Setting the force field gain higher than could be overcome fully by adjustments to limb impedance was desired in order to measure potential improvements in limb stability resulting from the application of tendon vibration.

Despite working in a force field half as strong as the control group, the stroke survivors did not respond by increasing their muscle activations and grip pressure as did control subjects. If stability in unstable environments is linked to co-contraction of arm musculature, it might seem intuitive that heightened reflex activity in the stroke arm [29] would generate beneficial stiffness that would help stroke patients to stabilize the arm while working in F_d_. However, when healthy subjects stabilize in such environments, the CNS uses a strategy of *controlling* viscoelastic impedance rather than a universal co-contraction of muscles throughout the arm [30]. In this manner, the CNS selectively increases stiffness corresponding to the direction and strength of the force field, while minimizing the level of stiffness in the direction of movement [27, 31]. The relatively poor performance by stroke subjects in this task may be due to an inability to use such control mechanisms for stability at the end of movement and might be attributed to already saturated muscle activity [32], the inability to consistently generate muscle forces [33], and pre-existing abnormal co-activation patterns in the arm [34]. Furthermore, the fact that across all trials, control subjects experienced higher error frequency at the endpoint suggests that they are indeed generating greater levels of endpoint stiffness than stroke subjects despite the stroke subjects’ disposition to heightened reflex activity.

Tendon vibration applied to the wrist flexors improved endpoint stability during forward arm movements in the divergent force field. While there were differences in stability error between groups, the effects of vibration appeared to be similar in both groups. Tendon vibration elicited a significant decrease in stability error at the end of forward arm movement for both stroke and control subjects and the error remained significantly lower after removal of vibration, suggesting that tendon vibration can be effective in enhancing feedback stabilization of the upper extremity at the end of reaching movements.

### Evaluating Mechanisms for Improved Stability

As previously discussed, co-contraction of antagonist muscles has been suggested as a common strategy for improving limb stability. Interestingly, increased co-contraction in response to either the force field or tendon vibration was not observed in stroke subjects as it was in control subjects (Fig 5) and therefore, increased co-contraction is not believed to be the mechanism by which stability was achieved for stroke survivors. While co-contraction in response to perturbation during movement (as observed in healthy subjects) may be a voluntary intervention to improve short-term stability during movement, it is likely that other subtle adaptations by the central nervous system could improve stability by altering reflex responses to resist perturbations or through small voluntary corrective responses aimed at slowing the movement over time [35]. In the present study and in similar prior studies [36], it is evident that stroke subjects indeed experience high levels of co-contraction in the muscle, even in baseline trials, indicating that modulation of co-contraction may simply not be an available response to the perturbation.

Modulation of joint damping via selective muscle activation has been identified as a second parameter potentially affecting limb stability [37]. At the wrist, increased damping corresponds with decreased oscillation velocity when adapting to negative viscosity [38, 39]. Perhaps in this study, with increased stiffness unavailable to the stroke survivors, a change in damping parameters is responsible for improved performance. The ability to make subtle corrective movements or changes in reflex activity could indeed increase damping at the end of movement and therefore decrease the oscillation of movement at the end of each trial during vibration. With this in mind, we must next evaluate the means by which subtle changes in reflex activity or corrective movements may be altered by vibration.

Previous studies have shown that sensory stimuli can improve motor functional performance for stroke patients. Electric stimulation of the ulnar and median nerves of the paretic hand applied for 120 minutes before a hand motor function test decreases the time required to complete the task post-stroke [40, 41]. Sustained functional improvements in response to combined stimulation and physical therapy are correlated with heightened cortical and corticomotor excitability produced by the electric stimulation [4, 42, 43]. Similar to electric stimulation, by increasing afferent firing rates, tendon vibration stimulates sensory afferent activity [8, 9] and is believed to be capable of increasing activity in both the somatosensory and motor cortex [17, 18, 44].

After vibration was removed in the current study, movements made in F_d_ maintained an improved stability, suggesting an adaptive response to the vibratory stimulus. TMS studies evaluating the ability of tendon vibration to enhance cortical output have demonstrated that within the range of 70–80 Hz, tendon vibration is capable of facilitating MEP amplitudes [10, 45, 46] and the augmented MEP response can be retained for up to 20 minutes after removal of the vibratory stimulus [11]. This evidence supports vibration’s ability to alter cortical activity beyond the stimulus duration and may explain improvements that were maintained in the post-vibration trials.

Another possible mechanism for improved stability due to vibration may directly relate to altering proprioceptive feedback to the CNS. One explanation for the improved stability with vibration is that augmented proprioceptive information may improve sensorimotor integration, allowing subjects to rely less on visual information for the planning and control of movement. To plan and execute a movement, the CNS typically uses a weighted combination of visual and proprioceptive information to estimate the arm’s position in space [47, 48]. The level of dependence on visual versus proprioceptive information is weighted based on movement conditions such as the sensory modality of the target [49, 50] and the relative reliability of visual and proprioceptive feedback of task performance [51]. The belief that proprioceptive information is critical for converting desired movement vectors into motor commands [52] suggests a lack of proprioception could play a large part in the stroke population’s inability to control secondary movements at the target. In the current study, a proprioceptive stimulus (wrist vibration) that was unrelated to the actual task (elbow and shoulder movement) improved stabilization. This raises the question of whether generic stimuli can improve sensory processing of information originating from the same modality (i.e. proprioceptive afferents).

After a stroke, impaired proprioception and tactile discrimination can limit the CNS’s ability to accurately detect the position of a limb in space [53, 54]. Although patients lacking proprioception are able to make isolated joint movements [55, 56], they are also known to experience directional errors during reaching [57], deficits in interjoint coordination of limb movements [58], impaired ability to stabilize the hand at a spatial target at the end of a reach [59] and an inability to maintain a specific joint angle without visual feedback [55, 56]. When available, visual information regarding limb posture is relied upon to reduce both direction and extent errors while reaching [60]. While the availability of visual information improves arm movement, relying solely on visual feedback may not be sufficient for ‘normal’ movement control.

Improvements in endpoint stability observed in the age-matched control group are believed to be related to similar mechanisms of enhancing sensorimotor integration. Older adults are known to experience depleted afferent sensory information. In fact, studies of elderly populations evaluating standing posture suggest postural instability is affected by the ability to integrate proprioceptive signals in the CNS [61, 62]. While sensory loss for healthy adults is minor compared to that lost by many chronic stroke individuals, our study indicates that enhancing proprioceptive information might improve steadiness for older adults during coordinated arm movements.

In our previous study, which evaluated the effect of tendon vibration on 8 directions of arm movement, improvements in stability were accompanied by a general decrease in muscle activity throughout the stroke arm [1]. In this experiment, the only significant effect of vibration on muscle activity was observed in the wrist flexors, where increased muscle activity occurred in both stroke and control subjects. This finding was consistent with previous observations that tendon vibration augments muscle forces in the homonymous muscle group [63]. Therefore, we believe vibration had a modest excitatory effect upon wrist flexor activity, with little overall effect on the baseline activity in other muscle groups.

It is possible that subtle changes in the timing and magnitude of muscle activity contributed to improved stability error in the B3 (Force + Vibration) condition as well as in the B4 (Force, Post-Vibration) condition. Given the challenges in making accurate measurements of shoulder muscle activity, it is possible that the EMG recordings of the current study were insufficient to detect small changes in EMG magnitude or (especially) timing. In addition, deeper muscles of the shoulder cannot be accurately quantified with the use of surface EMG. Another limitation of the current study is that the increased baseline level of muscle activation experienced by stroke subjects, coupled with the low sensitivity inherent in surface EMG, restricts the ability to capture subtle changes in muscle activation. This limitation likely impacted our ability to describe alterations of muscle activation timing that are triggered by vibration.

### Planar Movements in Proximal and Distal Workspaces

Despite prior reports that endpoint stability is less stable at distal target locations [64]; we observed no significant difference in stability error between trials at the proximal and distal targets for both subject groups in the present study. Similarly, muscle activity for the control subjects was not statistically different across distal and proximal regions in 6 of the 8 recorded muscles. In the control group, only AD and PD muscles exhibited differing levels of activity between workspace locations, with stability at the proximal target requiring relatively higher PD activity and the distal target requiring heightened AD activity. In contrast, stroke subjects demonstrated significantly higher levels of muscle activation in the distal workspace across all experimental conditions. Stroke survivors also demonstrated elevated grip pressure and lower error frequencies during trials conducted in the distal workspace.

Spasticity may partially explain the heightened muscle activity observed across muscle groups in the distal workspace. Particularly in the elbow musculature, spasticity is believed to increase as the joint is extended, resulting in higher reflex torques and elevated EMG activity in the musculature associated with elbow movement [29, 65]. In this experiment, moving to the distal target location required nearly full elbow extension while movement to the proximal target required a much lower change in joint angle. Accordingly, heightened levels of muscle activity in the distal workspace may be due to heightened spasticity inherent to reaching movements made toward the limits of the distal workspace.

### Clinical Significance

This study is clinically relevant because it suggests a therapeutic benefit of stimulating sensory afferents via tendon vibration to improve motor performance. Traditionally, post-stroke therapeutic interventions have focused on motor re-training. However, recent attention to the link between sensory impairments and motor deficits has raised interest in treatment of sensory dysfunction [46]. In people with somatosensory deficits, improvements in proprioceptive and tactile impairments are associated with improved functional outcomes [66]. Specifically related to proprioceptive sense, the therapeutic use of repetitive passive arm movement has been shown to stimulate brain sensorimotor activity [67] and the incorporation of proprioceptive and tactile training can be used therapeutically to elicit improvements in functional tasks [68]. The current study provides a real-time method for stimulating proprioceptive afferents to the CNS and demonstrates short-term improvements in endpoint stability. Future studies are necessary to evaluate the effect of such therapies on functional tasks and to identify the specific supraspinal mechanisms contributing to functional improvement.

## Supporting Information

S1 Dataset Raw endpoint stability, EMG and grip data for stroke and control subjects. (XLSX)
